# Acceptability and preferences of people with long-term conditions for delivery of digital healthcare interventions: scoping review protocol

**DOI:** 10.1136/bmjopen-2024-095798

**Published:** 2025-08-12

**Authors:** Charlotte Gerlis, Alice Berry, Rachel Thomas, Serena Pacey-halls, Michael Loizou, Caroline Swales, Fiona Cramp

**Affiliations:** 1 University of the West of England, Bristol, UK

**Keywords:** Chronic Disease, Digital Technology, eHealth

## Abstract

**Abstract:**

**Background:**

Digital health interventions (DHIs) are prevalent and have been shown to help some people with long-term conditions (LTCs) to manage their condition. There are myriad options for digital delivery yet limited understanding of what modes of delivery are acceptable to people with LTCs. It is important to understand the acceptability of delivery methods of DHIs to inform future DHI development and promote engagement. This scoping review aims to explore the acceptability of the delivery of DHIs for people with LTCs.

**Methods and analysis:**

This review will follow the Joanna Briggs Institute guidance for scoping reviews and will be reported in line with the Preferred Reporting Items for Systematic Reviews and Meta-Analyses Scoping reviews extension checklist. Databases including MEDLINE, PubMed, CINAHL, AHMED and PsycINFO will be searched for primary studies that provide data on preferences for delivery methods of DHIs by people with LTCs. Narrative analysis is anticipated, and a summary of the findings will be presented in a tabulated format.

**Ethics and dissemination:**

Ethical approval will not be required for this scoping review. The findings will be disseminated via appropriate peer-reviewed journals and conferences and PhD theses.

**STRENGTH AND LIMITATIONS OF THIS STUDY**

- Broad inclusion criteria to explore an under-researched area of digital health interventions (DHIs).
- Following the Joanna Briggs Institute protocol for scoping reviews and the Preferred Reporting Items for Systematic Reviews and Meta-Analyses framework to ensure key areas of the scoping review are reported.
- Although not required for a scoping review, sources of evidence included in the scoping review will be critically appraised, adding to understanding of its reliability, importance and applicability
- Only studies available in the English language will be included.

## Introduction

### Background and rationale

 People with long-term conditions (LTCs) have a health problem that cannot be cured and requires on-going management with medication or other therapies.^1 2^ This population is increasing and places a substantial burden on the National Health Service and other care services.^3 4^ Recently, there has been a significant reduction in health-related quality of life and in the number of people with LTCs who feel supported to manage their condition.^5^ A need to focus on improving outcomes for people with multiple LTCs was also highlighted.^5^ Innovation in digital health to develop and implement new interventions is rapid and provides new ways to deliver and support effective healthcare.^6^ Digital health interventions (DHIs) are a key solution to address the increasing demands on health systems.^7^

Digital healthcare describes the general use of information and communication technologies for health and is inclusive of both mHealth (the use of mobile and wireless technologies for health) and electronic health.^8^ Increasingly, health interventions to manage LTCs are being delivered digitally. DHIs are a flexible, widely accessible and cost-effective support option that can be tailored to individuals.^9^ DHIs have shown promising outcomes across LTCs including rheumatoid arthritis, cardiovascular disease and chronic kidney disease.^10^,^12^ There are many types of DHI function ranging from system impact to understanding and communication and interventions.^6^

There are myriad options for how a health intervention is delivered digitally. An extensive array of electronic platforms could host a DHI. Such platforms can be categorised into internet and non-internet based. Non-internet based platforms include mobile phones (such as short message service text, voice communication and audio or video clips).^13^ Internet-based platforms include smart phones, computers, augmented reality and 3D virtual reality.^14 15^ Digital healthcare intervention delivery provides opportunity for variety in other aspects of delivery. Delivery may be synchronous, with information being delivered in real time (eg, video or telephone calls) or asynchronous (eg, information delivered by discussion boards or email). The level of interactivity may vary from unidirectional, where the causal influence is from the intervention source, to interactional between intervention and recipient.^16 17^ There may be a single recipient or a group, and the intervention source may be non-human or human such as healthcare professionals or peers.^15^

There is a significant variety in the potential options for delivering a DHI. The mode of delivery is known to be an important component of DHIs that aim to change behaviour. Mode of delivery can enhance or undermine intervention effectiveness, influencing outcomes.^16 17^ Despite this, the delivery of behaviour change interventions has received much less attention compared with the intervention content.^16^ Given that mode of delivery can enhance or undermine intervention effectiveness, it is important to understand what is desirable, usable and wanted by their intended users.^16 17^

More broadly, there is limited literature exploring the preferences and acceptability of people with LTCs for how DHIs are delivered. Acceptability reflects the extent to which people using the intervention consider it to be appropriate, based on experienced cognitive or emotional responses to it.^18^ Acceptability is a complex, dynamic process which moves through prospective (pre-use), concurrent (during use) and retrospective (after use) constructs.^19^ The distinction between anticipated acceptability (prospective or preference) and experienced acceptability (during or after use) is a key feature of the theoretical framework of acceptability. Assessment of prospective acceptability can highlight aspects of an intervention that could be modified to increase acceptability.^18^ Acceptability is a key feature in the UK Medical Research Council framework for developing complex interventions which highlights the importance of meaningful engagement with key stakeholders, to facilitate implementation and practicability of the intervention.^20^ An intervention with a delivery method that is not acceptable to intended users will limit uptake, adoption and use.^17^

The COVID-19 pandemic led to pragmatic and quick adoption of DHIs to deliver healthcare remotely. This was despite previous concerns that DHIs were being developed without input from the service users. The rapid increase in use of DHIs during the pandemic was widely accepted by patients in the context of social distancing requirements.^21^ However, it highlighted the digital divide where some patient groups were not willing or able to make use of DHIs.^21^ In 2019, it was reported that 9% of the UK adult population were non-internet users compared with 23% of disabled adults. Therefore, it is likely that non-internet DHIs (eg, short message service texts, telephone calls, diary reminders) are preferable for some people with LTCs.^22^ Older adults from ethnic minority backgrounds and socioeconomically disadvantaged groups, who are at greater risk of multiple LTCs, have low utilisation of the internet for health information.^23 24^ Internet usage has also been shown to vary across UK regions.^22^ To minimise the risk of digital health inequalities, it is important to understand preferences for DHI delivery of people with LTCs.

It is expected that an intervention where acceptability of mode of delivery has been considered is likely to have improved engagement and accessibility for its intended users. Therefore, this scoping review will focus on the population of people with LTC, to explore the concept of acceptability and preference in the context of DHI modes of delivery. This work will be important to support refinement of existing interventions and support future DHI development.

#### Research questions

This scoping review aims to answer the following question, ‘What mode of delivery do people with LTCs find acceptable or preferable for DHIs?’

#### Review aims

The aim of this scoping review is to provide a broad overview of the evidence exploring what people with LTCs find acceptable or preferable for the delivery of DHIs.

The objectives of this scoping review are:

- To identify and map the existing evidence exploring acceptability and preferences for digital delivery of DHIs in populations with LTCs.
- To explore whether there are any gaps in literature across different LTCs or subgroups of the populations with LTCs.
- To provide recommendations for the delivery of future development of DHIs for people with LTCs

## Methods

This scoping review will be conducted in accordance with the Joanna Briggs Institute (JBI) methodology for scoping reviews which provides a comprehensive framework for conducting a scoping review.^1^ Alongside this, the ‘Preferred Reporting Items for Systematic Reviews and Meta-Analyses extension for Scoping Reviews (PRISMA-ScR)’ has been used to inform this reporting and will be used to structure the full review. This will ensure good quality methodology and that key items for the scoping review are reported.^25^ The initial searches were conducted in September 2022; this research is expected to end by October 2025.

### Eligibility criteria

The following eligibility criteria for the inclusion of papers was based on participants, concept and context. Due to the broad nature of these search terms and lack of homogenous definitions for acceptability, digital modes of delivery and DHIs, the authors anticipate refining the search criteria following a first round of screening.

#### Participants

Participants with a LTC who are 18 years old or over. A LTC will be defined as a condition for which there is currently no cure. Where additional participants are recruited, the data must be reported separately for adults with LTCs.

#### Concept

Primary studies that explore preferences and acceptability for delivery methods of DHIs. These may include digital platform, synchronicity, interactivity and the person or method of delivering the intervention.

#### Context

Studies that explore preferences for delivery of DHIs. Interventions may include self-management, monitoring, consultations and rehabilitation interventions.

### Information sources

This scoping review will consider both experimental and quasiexperimental study designs including randomised controlled trials, non-randomised controlled trials, before and after studies and interrupted time-series studies. In addition, analytical observational studies including prospective and retrospective cohort studies, case-control studies and analytical cross-sectional studies will be considered for inclusion. This review will also consider descriptive observational study designs including case series, individual case reports and descriptive cross-sectional studies for inclusion. Policy papers and digital health programmes that meet the criteria will be included.

Qualitative studies will also be considered that focus on qualitative data including, but not limited to, designs such as phenomenology, grounded theory, ethnography, qualitative description, action research and feminist research.

### Search strategy

The search strategy will aim to locate both published and unpublished studies. An initial limited search of PubMed and CINAHL was undertaken to identify articles on the topic. The text words contained in the titles and abstracts of relevant articles, and the index terms used to describe the articles were used to develop a full search strategy for MEDLINE, PubMed, CINAHL, AHMED and PsycINFO (online supplemental appendix 1). Only studies published in the English language will be included as resources for accurate translation are not available. Where abstracts and full texts are not in the English language, a translated version will be sought. There will not be any date ranges imposed. Following full text inclusion, reference lists of included studies will be screened for additional relevant studies. Identified articles in forward citation searching will also be assessed for relevance. The sources of unpublished studies/grey literature to be searched include trial registers such as ISRCTN, British Library EThOS and Overton.

### Selection of sources of evidence

Following the search, all identified citations will be collated and uploaded into the bibliographic software Rayyan and duplicates removed. Following a pilot test, titles and abstracts will then be screened by the lead author (CG) and one other independent reviewer (FC, AB or RT) for assessment against the inclusion criteria for the review. Citations will only be excluded where both reviewers independently identify them as not relevant. All remaining sources will be retrieved in full, and their citation details will be imported into Rayyan. The full text of selected citations will be assessed in detail against the inclusion criteria by two independent reviewers. Reasons for exclusion of sources of evidence at full text that do not meet the inclusion criteria will be recorded and reported in the scoping review. Any disagreements that arise between the reviewers at full text stage will be resolved through discussion, and if necessary, with input from an additional reviewer/s. The results of the search and the study inclusion process will be reported in full in the final scoping review and presented in a PRISMA-ScR flow diagram.^2^

### Data extraction

Data will be extracted from the included papers by one reviewer and checked by another using a version of the JBI template for data extraction tool developed specifically for the review. The data extracted will include details about the participants, the DHI, the mode of delivery (eg, platform, synchronicity, interactivity), study methods and other key findings relevant to the review questions. A draft extraction form will be piloted using three included studies. If there is sufficient breadth and volume of DHIs identified, the characteristics of the studies will be mapped using the WHO Classification of digital interventions, services and applications in health (2023).

The data extraction tool will be modified and revised as necessary during the process of extracting data from each included evidence source. Any disagreements that arise between the reviewers will be resolved through discussion or with an additional reviewer. If appropriate, authors of the papers will be contacted to request additional data or to seek clarification.

A draft of the extraction tool, based on the JBI tool, is provided (see online supplemental appendix 2). The draft data extraction tool will be modified and revised as necessary during the process of extracting data from each included evidence source. Modifications will be detailed in the scoping review. Any disagreements that arise between the reviewers will be resolved through discussion, or with an additional reviewer/s. Where required, authors of papers will be contacted to request missing or additional data.

### Critical appraisal of individual sources of evidence

Although not required for a scoping review, the included sources of evidence will be critically appraised using the standardised JBI critical appraisal tool appropriate for the study design.^1^ This will improve understanding of the reliability, applicability and importance of the included evidence.

### Synthesis of results

Findings of the study will be presented in a PRISMA diagram, highlighting the number of articles identified, their sources and reasons for exclusion and full text-screening.^2^ All included articles will be summarised in a table. Due to the expected varied range of study types being included, data analysis will likely be narrative. Due to the nature of the review, it is anticipated that qualitative studies will be included which will be analysed using inductive content analysis.^26^ It is anticipated that a summary of the findings will be presented in a tabular format.

## Patient and public involvement

This research contributes to a larger PhD project. A patient research partner has been involved from the beginning of the project, including funding application and research design. The patient research partner will be invited to comment on the review findings. This review will contribute to the codevelopment of a digital behaviour change intervention to promote physical activity.

## Ethics and dissemination

Ethical review and approval is not required for this scoping review. The review findings will be disseminated through appropriate peer-reviewed journals, conference presentations and PhD theses.

## Supplementary material

10.1136/bmjopen-2024-095798 online supplemental file 1

10.1136/bmjopen-2024-095798 online supplemental file 2
