# Supplementary material for: Acceptability and preferences of people with long-term conditions for delivery of digital healthcare interventions: scoping review protocol
Source: BMJ Open. 2025 Aug 12;15(8):e095798. doi: 10.1136/bmjopen-2024-095798 (PMC12352148; doi:10.1136/bmjopen-2024-095798)
Supplement: online supplemental file 1 [file bmjopen-15-8-s001.docx]

## Appendix 1

### Example search strategy:

*‘long term condition’ OR ‘long term disease’ OR ‘chronic condition’ OR ‘chronic disease’ OR ‘chronic illnesses’ OR ‘long term illness’*

*AND*

*‘remote’ OR ‘virtual’ OR ‘digital’ OR ‘tele’ OR ‘telehealth’ OR ‘online’ OR ‘mHealth’ OR ‘internet’ OR ‘technology’ OR ‘eHealth’ OR ‘mobile’ OR ‘remote’*

*AND*

*‘deliver*’ OR ‘provision’ OR ‘provide’ OR ‘features’ OR ‘components’*

*AND*

*‘preference*’ OR ‘choice*’ OR ‘prospective acceptability’ OR ‘discrete choice experiment*’OR ‘acceptability’*
