# Supplementary material for: Acceptability and preferences of people with long-term conditions for delivery of digital healthcare interventions: scoping review protocol
Source: BMJ Open. 2025 Aug 12;15(8):e095798. doi: 10.1136/bmjopen-2024-095798 (PMC12352148; doi:10.1136/bmjopen-2024-095798)
Supplement: online supplemental file 2 [file bmjopen-15-8-s002.docx]

## Appendix 2

A draft data extraction table

|  | **Study Number** | **1** | **2** | **3** |
| --- | --- | --- | --- | --- |
| **Study information** | Author(s) |  |  |  |
|  | Title |  |  |  |
|  | Date |  |  |  |
|  | Country |  |  |  |
|  | Type study (quantitative, quantitative and mixed methods) |  |  |  |
|  | Methodology |  |  |  |
|  | Findings |  |  |  |
| **Participants** | Number of participants |  |  |  |
|  | Long Term Condition(s) |  |  |  |
|  | Gender |  |  |  |
|  | Ethnicity |  |  |  |
|  | Other relevant characteristics |  |  |  |
|  | Setting |  |  |  |
| **Interventions** | Mode of delivery |  |  |  |
|  | Length |  |  |  |
|  | Purpose |  |  |  |
|  | Content |  |  |  |
|  | Author conclusion |  |  |  |
|  | Reviewer comments |  |  |  |
