# Supplementary material for: Acceptability and preferences of people with long-term conditions for delivery of digital healthcare interventions: scoping review protocol
Source: BMJ Open. 2025 Aug 12;15(8):e095798. doi: 10.1136/bmjopen-2024-095798 (PMC12352148; doi:10.1136/bmjopen-2024-095798)
Supplement: Supplementary file 3 [file bmjopen-2024-095798.reviewer_comments.pdf]

PEER REVIEW HISTORY

BMJ Open publishes all reviews undertaken for accepted manuscripts. Reviewers are asked to complete a checklist review form and are provided with free text boxes to elaborate on their assessment. These free text comments are reproduced below.

ARTICLE DETAILS

Title (Provisional)

Acceptability and preferences of people with long-term conditions for delivery of digital healthcare interventions: scoping review protocol

Authors

Gerlis, Charlotte; Berry, Alice; Thomas, Rachel; Pacey-halls, Serena; Loizou, Michael; Swales, Caroline; Cramp, Fiona

---

VERSION 1 - REVIEW

**Reviewer**	**1**
**Name**	**Gunasekeran, Dinesh Visva**
**Affiliation**	**Singapore General Hospital**
**Date**	**21-Nov-2024**
**COI**	**None**

This would be an informative review and has been designed with reasonable methods. However in its current form, it would benefit from some further refinement;

1. Intervention and study group being investigated are not well-characterised, and the search terms particularly for LTC are likely to miss out several key categories of patients/clinical contexts that otherwise fall in the scope of this review.

See examples:

A. https://www.nature.com/articles/s41746-021-00412-9

B. https://www.thelancet.com/journals/landig/article/PIIS2589-7500(20)30287-9/fulltext

2. The authors characterise the topic as an under-researched area, however there have been significant earlier reviews of related/adjacent topics such as those above that could be informative for this field, and may provide potential ways to refine methods eg characterising the DHIs. The authors could improve the abstract/introduction by discussing relevant major reviews and identifying gaps they hope to address with this work.

3. A minor suggestion - aside from content analysis displayed in a tabular format as indicated by the authors in their plan, perhaps some interesting figures might be useful for readers to

get an overview of the scale of literature for specific LTC topics. OCEBM construct (used in 1A above) could be applied to generate such diagrams from tabular data, or content analysis could be depicted with icons for this purpose.

**Reviewer**	**2**
**Name**	**Balappanavar, Aswini Y**
**Affiliation**	**Maulana Azad Institute of Dental Sciences**
**Date**	**07-May-2025**
**COI**	**None**

I would like to commend the authors on a well-written and methodologically robust protocol. The manuscript is a timely and well-rounded strategy for investigating the acceptability and preferences of individuals with long-term conditions for digital delivery of healthcare. The wide scope and strict adherence to the Joanna Briggs Institute and PRISMAScR guidelines are worthy of praise. Nevertheless, the study can be made even more robust by responding to the following suggestions:

Terminological Clarity

The use of the words "digital interventions," "digital health," "digital technology," and "digital delivery" seems interchangeable across the paper. For clear conceptual purposes and to avoid any confusion, authors are advised to incorporate more repetitive definitions
or might find it prudent to refer to already existing digital health taxonomies (e.g., WHO Digital Health Classification) in order to facilitate the categorization of the words.

Explicit Research Question

Although the objectives and aims are well set out, a specific research question must be explicitly stated to ground the review and enhance reader clarity.

Inclusion of PCC Framework in Introduction

Including the Population, Concept, and Context (PCC) framework in the Introduction section would be beneficial to more effectively organize the rationale and align it with typical scoping review approaches.

Enlarged Objectives

Authors could consider including an objective describing how conclusions will guide a roadmap or recommendations for future development of digital interventions, especially as responses to gaps in the existing literature.

Language Limitations

Although limiting to English language studies is reasonable, an explanation of why and how non-English studies might be included (with possible translation assistance) would reduce language bias and make the findings more internationally relevant.

Policy and Programmatic Sources

Explanation of whether policy papers or digital health programs will be included in the scoping review is necessary. Such documents are included to facilitate wider understanding about the acceptability of digital interventions from the views of decision-makers and stakeholders taking part in providing healthcare.

Grey Literature and Unpublished Evidence

The protocol does specify inclusion of some grey literature (such as clinical trial registries), but it would be advantageous to indicate if other types of grey or unpublished literature (such as white papers, government documents, dissertations) are included as well since they might contribute value to under-studied areas.

Would digital access disparities be considered within the framework of accessibility?

Disclosure: Chatgpt 4.0 has been used for grammar correction and paraphrasing

---

VERSION 1 - AUTHOR RESPONSE

Reviewer: 1

1. Intervention and study group being investigated are not well-characterised, and the search terms particularly for LTC are likely to miss out several key categories of patients/clinical contexts that otherwise fall in the scope of this review.

The search terms for long-term conditions have been reviewed by the authors and a subject support librarian and considered to be appropriate to our review aim. We considered expanding the terms to include specific long-term conditions but have not identified an exhaustive list.

2. The authors characterise the topic as an under-researched area, however there have been significant earlier reviews of related/adjacent topics such as those above that could be informative for this field, and may provide potential ways to refine methods eg characterising the DHIs. The authors could improve the abstract/introduction by discussing relevant major reviews and identifying gaps they hope to address with this work.

It has been interesting and informative to read the above work in the area of ophthalmology and digital health solutions such as AI decision support for triage and clinical care and home monitoring. Given the large scope of digital health interventions that may be used for people with long-term conditions we

anticipate a possible need to conduct a second title and abstract screen, limiting to such non-medical digital health interventions, depending on the volume of included titles and abstracts initially included. Whilst it is beyond our scope to characterise all the DHIs identified in the literature for people with LTCs, we will map the characteristics of the included DHIs and have elaborated on this in lines 188-191.

3. A minor suggestion - aside from content analysis displayed in a tabular format as indicated by the authors in their plan, perhaps some interesting figures might be useful for readers to get an overview of the scale of literature for specific LTC topics. OCEBM construct (used in 1A above) could be applied to generate such diagrams from tabular data, or content analysis could be depicted with icons for this purpose.

Thank you for this suggestion, we will consider this at the point of data analysis and write up.

Reviewer: 2
Terminological Clarity

The use of the words "digital interventions," "digital health," "digital technology," and "digital delivery" seems interchangeable across the paper. For clear conceptual purposes and to avoid any confusion, authors are advised to incorporate more repetitive definitions or might find it prudent to refer to already existing digital health taxonomies (e.g., WHO Digital Health Classification) in order to facilitate the categorization of the words.

The use of these terms across the paper have been reviewed and amended to improve clarity. In line with the WHO Digital Health Classification the term 'digital health interventions' has been used. The term digital delivery has still been used in some cases to refer to delivery as a specific aspect of a digital health intervention.

Explicit Research Question

Although the objectives and aims are well set out, a specific research question must be explicitly stated to ground the review and enhance reader clarity.

A research question based on the PCC framework has been included (line 107-109).

Inclusion of PCC Framework in Introduction
Including the Population, Concept, and Context (PCC) framework in the Introduction section would be beneficial to more effectively organize the rationale and align it with typical scoping review approaches.

A section at the end of the introduction has been rephrased to make the population, concept and context clear (lines 102-104).The introduction has been organised as population, then context (digital health interventions and the myriad options for delivery), and then concept (acceptability and preference). It felt important to introduce the context of digital health interventions and delivery methods first as this less-researched area forms part of the rationale for exploring acceptability and preferences.

Enlarged Objectives
Authors could consider including an objective describing how conclusions will guide a roadmap or recommendations for future development of digital interventions, especially as responses to gaps in the existing literature.

An aim has been added to consider this (line 118 – 119).

Language Limitations

Although limiting to English language studies is reasonable, an explanation of why and how non-English studies might be included (with possible translation assistance) would reduce language bias and make the findings more internationally relevant.

As resources are limited it will not be possible to translate non-English texts, but the process of identifying texts in the English language has been expanded on (line 161-163).

Policy and Programmatic Sources
Explanation of whether policy papers or digital health programs will be included in the scoping review is necessary. Such documents are included to facilitate wider understanding about the acceptability of digital interventions from the views of decision-makers and stakeholders taking part in providing healthcare.

We have included a statement to indicate that policy and digital health programs will be included, if they include the views of adults with LTCs (line 151-152). The authors considered including acceptability and preferences of stakeholders (e.g. health care professionals, carers) and decision makers but felt this would make the review too broad.

Grey Literature and Unpublished Evidence
The protocol does specify inclusion of some grey literature (such as clinical trial registries), but it would be advantageous to indicate if other types of grey or unpublished literature (such as white papers, government documents, dissertations) are included as well since they might contribute value to under-studied areas.

The database Overton will also be searched to identify a wider range of grey literature, line 167 has been amended to specify this.

Would digital access disparities be considered within the framework of accessibility?

Digital access disparities are not specifically considered within the framework. This is also an important area to explore, but we have not included this as an outcome as it is beyond the scope of this review.
